# Development and validation of a short version of the Partnership Self-Assessment Tool (PSAT) among professionals in Dutch disease-management partnerships

**DOI:** 10.1186/1756-0500-4-224

**Published:** 2011-06-30

**Authors:** Jane M Cramm, Mathilde MH Strating, Anna P Nieboer

**Affiliations:** 1 Institute of Health Policy & Management (iBMG), Erasmus University, Rotterdam, The Netherlands

**Keywords:** chronic care, measurement, quality, chronic illness, health care, partnership synergy, isease management

## Abstract

**Background:**

The extent to which partnership synergy is created within quality improvement programmes in the Netherlands is unknown. In this article, we describe the psychometric testing of the Partnership Self-Assessment Tool (PSAT) among professionals in twenty-two disease-management partnerships participating in quality improvement projects focused on chronic care in the Netherlands. Our objectives are to validate the PSAT in the Netherlands and to reduce the number of items of the original PSAT while maintaining validity and reliability.

**Methods:**

The Dutch version of the PSAT was tested in twenty-two disease-management partnerships with 218 professionals. We tested the instrument by means of structural equation modelling, and examined its validity and reliability.

**Results:**

After eliminating 14 items, the confirmatory factor analyses revealed good indices of fit with the resulting 15-item PSAT-Short version (PSAT-S). Internal consistency as represented by Cronbach's alpha ranged from acceptable (0.75) for the 'efficiency' subscale to excellent for the 'leadership' subscale (0.87). Convergent validity was provided with high correlations of the partnership dimensions and partnership synergy (ranged from 0.512 to 0.609) and high correlations with chronic illness care (ranged from 0.447 to 0.329).

**Conclusion:**

The psychometric properties and convergent validity of the PSAT-S were satisfactory rendering it a valid and reliable instrument for assessing partnership synergy and its dimensions of partnership functioning.

## Background

The prevalence of individuals with chronic illness is growing fast because of the rapid aging of the population and the greater longevity of individuals. The multiple and often complex needs of populations affected by the epidemic of chronic illnesses requires approaches that include collaboration among health care professionals of various organizations and extend beyond traditional acute episodic health care and the services of any single organization [1-5]. Partnerships are increasingly used to enhance health service delivery in response to this explosion in chronic disease prevalence. While interprofessional health partnerships are internationally acknowledged as integral for comprehensive chronic illness care, the evidence for effectiveness of such partnerships is lacking [6,7]. Partnership collaboration requires relationships, procedures, and structures that are quite different from the ways many people and organizations have worked in the past and research indicates that these partnerships are also generating a good deal of frustration [8]. In addition, building effective partnerships is time consuming, resource intensive, and often very difficult [9-12]. A number of special challenges are involved in the management of interprofessional health partnerships [12,13]. One of the main critical tasks of partnership management is to enhance the capacity of partnerships to achieve high levels of synergy. Partnership's ability to create synergy resulting in the redesign of patient care is an essential factor for successful disease-management [7,8]. Synergy is the degree to which the partnership combines the complementary strengths, perspectives, values and resources of all partners in the search for better solutions [14: p. 5] and is generally regarded as the product of a partnership [15]. The synergy that a partnership can achieve is more than simply an exchange of resources among its partners. Theoretically, when partners effectively merge their perspectives, knowledge, and skills to create synergy, they create something new and valuable: a whole that is greater than the sum of its parts. Lasker and colleagues [8] developed a framework that supports the people responsible for managing partnerships in realizing high levels of synergy. The Partnership Self-Assessment Tool (PSAT) was developed based on this framework by public health specialists for practical use by groups working to promote health and well-being in their communities [7]. It measures partnership synergy and other related dimensions of the partnership process [16]. Establishment of construct validity during development of the PSAT items was rigorous [7,9]. In addition, it included data from qualitative interviews with members of community health promotion partnerships, an extensive review of relevant literature and measures, as well as input from a panel of experts. Furthermore, the PSAT was tested in 63 health-related partnerships in operation at least 18 months in urban, suburban or rural areas in the US [7]. A recent study conducted by Butt and colleagues [17] showed that the PSAT is a valid partnership process measurement tool.

The PSAT has not been validated in the Netherlands to date. In this article, we describe the psychometric testing of the PSAT among professionals in twenty-two disease-management partnerships participating in quality improvement projects focused on chronic care in the Netherlands. Our objectives are to validate the PSAT in the Netherlands and to reduce the number of items of the original PSAT while maintaining validity and reliability.

## Methods

### Disease-management partnerships

The study is in the context of a national programme on "disease management of chronic diseases" carried out by ZonMw (Netherlands Organisation for Health Research and Development) and commissioned by the Dutch Ministry of Health. The study was approved by the ethics committee of the Erasmus University Medical Centre of Rotterdam (September 2009).

This study is focused on the evaluation of the implementation of twenty-two disease-management partnerships to enhance knowledge on disease-management experiments in chronic disease care, and stimulate implementation of knowledge and insights of successful programs. These disease-management partnerships are located in various Dutch regions and consist of a variety of collaborations between organizations and/or professionals, e.g. collaborations between general practices and hospitals, primary care collaborations (including physiotherapists and dieticians) or primary and community settings. A questionnaire was sent to all 393 professionals participating within the 22 disease-management partnership (consisting of 153 organizations). Either a package of questionnaires was sent to the contact person of each participating organization (which were distributed to potential respondents through their mail boxes or delivered personally at team meetings) or questionnaires were sent directly to the potential respondents. Two weeks later the same procedure was used to send a reminder to non-respondents. A total of 218 respondents filled in the questionnaire (55% response rate; range 35-100%).

The disease-management partnerships are targeted at different patient populations; including cardiovascular diseases (9 disease-management partnerships), chronic obstructive pulmonary disease (COPD) (5), diabetes (3), heart failure (1), stroke (1), depression (1), psychotic diseases (1) and eating disorders (1). The intervention concerns the implementation of disease-management partnerships aiming to improve quality of chronic care based on the Chronic Care Models (CCM). Each disease-management partnership consists of a combination of patient-related, professional-directed and organizational interventions. The exact programme components for each region may vary. The core of a DMP is described below; for detailed programme information, see our study protocol [18].

#### Patient-related interventions

All 22 disease-management partnerships implemented self-care interventions e.g. patient education on lifestyle, regulatory skills, and proactive coping.

#### Professional-directed interventions

Care standards, guidelines, and protocols are essential parts of the 22 disease-management partnerships. They are integrated through timely reminders, feedback, and other methods that increase their visibility at the time that clinical decisions are made. The disease-management partnerships are built on these (multidisciplinary) guidelines. The implementation strategies for professional interventions may, however, vary. All disease-management partnerships provide training for their professionals and implementation of these guideline is supported in almost all disease-management partnerships by ICT tools such as integrated information systems.

#### Organisational interventions

Many forms of organisational changes are applied in the 22 disease-management partnerships e.g. new collaborations of care providers, allocating tasks differently, transferring information and scheduling appointments more effectively, case management, using new types of health professionals, redefining professionals' roles and redistributing their tasks, planned interaction between professionals, and regular follow-up meetings by the care team.

### Measures

The PSAT was used to measure partnership synergy and dimensions of partnership functioning [7,8]. This instrument contains partnership synergy (nine items e.g. by working together, how well are these partners able to identify new and creative ways to solve problems) and four dimensions of partnership functioning: leadership (11 items e.g. fostering respect, trust, inclusiveness, and openness in the partnership), efficiency (three items e.g. how well your partnership uses the partners' financial resources), administration and management (nine items e.g. evaluating the progress and impact of the partnership), and resources (six items e.g. data and information: statistical data, information about community perceptions, values, resources, and politics). Responses to all items are structured by a five-point Likert scale.

Partnership synergy and dimensions of partnership functioning scores were derived by calculating the mean of responses within each section. The partnership synergy score reflects the extent to which the participants in the disease-management partnership are accomplishing more together than they can on their own [7].

Since the disease-management partnerships aim to improve quality of chronic care based on the CCM, we tested convergent validity with the Assessment of Chronic Illness Care (ACIC). The ACIC consists of 34 items covering the seven areas of the CCM: health care organization (6 items); community linkages (3); self-management support (4); delivery system design (6); decision support (4); clinical information systems (5). The ACIC also covers integrating the six components, such as linking patients' self-management goals to information systems (6 items) [19]. Subscale scores for the seven sections of the ACIC were derived by calculating the mean of responses within each section, and overall scores were derived by calculating the mean of all subscale scores. We used overall scores to assess the effectiveness of disease-management partnership in delivering chronic-illness care. The highest possible score (11) for any individual item, subscale, or overall score was taken to indicate optimal chronic-illness care delivery, and the lowest score (0) was considered to correspond with limited chronic illness care delivery.

### Analyses

The analyses included the following six steps.

1. The sample characteristics were analysed using descriptive statistics.

2. We data-screened the items by examining number of missing, number of 'don't know' answers, mean and standard deviation of each item.

3. To verify the factor structure of the questionnaire and to test whether the relationship between observed variables and their underlying latent constructs exists, confirmatory factor analysis was executed using the LISREL program [20]. In line with the theoretical assumptions of the measurement model, no correlation errors either within or across sets of items were allowed in the model.

4. Item reduction analysis was performed to develop a short version of the questionnaire that can be used in case the original version is considered to be too long. Items were removed from the original pool following several criteria: 1) Items were excluded one by one, first by eliminating items with factor loadings below .40 and second by following the modification indices, 2) eliminating items was stopped when reliability (Cronbach's alpha) of each subscale drops below .70 and 3) there should be as few items as possible with a minimum of three, without loss of content and psychometric quality. Listwise deletion of cases with missing data on these 29 items resulted in N = 68. Imputation was done by replacing missing values by the mean of each disease-management partnership as scored by the other professionals of the same disease-management partnership, resulting in N = 218 which was the total sample. To test the measurement models four indices of model fit were used. The cut-off criteria for these four indices were those proposed by Hu and Bentler [21]. First, the overall test of goodness-of-fit assesses the discrepancy between the model implied and the sample covariance matrix by means of a normal-theory weighted least squares test. A plausible model has low, preferably non-significant χ^2 ^values. However, Chi-square is overly sensitive when the sample size is large (anything over 200) [22], leading to difficulty in obtaining the desired non-significant level [23]. Secondly, the Root Means Square Error of Approximation (RMSEA) reflects the estimation error divided by the degrees of freedom as a penalty function. Values on RMSEA below cut-off value 0.06 indicate small differences between the estimated and observed model. Thirdly, we used the Standardized Root Means square Residual (SRMR), which is a scale invariant index for global fit that ranges between 0 and 1. Values on SRMR lower than cut-off value 0.08 indicate a good fit. As a fourth index of model fit the Incremental Fit Index (IFI) was calculated. This index compares the independence model (i.e. observed variables are unrelated) to the estimated model. Preferably, values on IFI should be larger than cut-off value 0.95.

5. Internal consistency of the subscales was assessed by calculating Cronbach's alphas, inter-item correlations within each subscale and correlations between subscales.

6. We then investigated convergent validity of the instrument. Validity reflects the degree to which a scale measures what it is intended to measure. We evaluated convergent validity by analyzing the associations between the dimensions of partnership (original instrument and short version) with partnership synergy and the ACIC.

## Results

### Sample characteristics

The response rate of the professionals was 55% (218/393). The majority of the professionals that filled in the baseline questionnaire were female (66.2%). Mean age was 47.2 years (sd 9.47) ranging from 25 to 65. Table 1 lists descriptive characteristics of the sample of professionals. The majority of the professionals (75.1%) had been working for more than 3 years within the organisation. Furthermore, 144 (67.6%) professionals worked more than 29 hours per week. Disease-management partnerships mainly consisted of general practitioners (34.9%) and practice nurses (25.7%). Other professionals participating in the disease-management partnership are paramedical professionals (11.9%) and medical specialists (2.8%).

**Table 1:** Sample characteristics professionals (n = 218)

		N	Percentage
Gender	- female	139	66.2%
	- male	71	33.8%
Working past	- more than 3 years	160	75.1%
Working hours per week	- more than 29 hours	144	67.6%
Occupation	- General Practitioner	76	34.9%
	- practice nurses	56	25.7%
	- policy and management	28	12.8%
	- para-/perimedical professionals	26	11.9%
	- medical/social specialists	6	2.8%
	- others	26	11.9%

### Datascreening

All items were screened for univariate and bivariate normality, and to detect outliers. Data screening information was taking into account in the stepwise procedure of the item reduction analysis. In general, percentages missings and 'don't know' answers were below 10%, except for item 8 and 9 of the leadership subscale and all of the items for administration and management subscale (see Table 2). Especially, the items within the administration and management subscales had relatively high percentages of 'don't know' answers.

**Table 2:** Item characteristics and factor loadings of the first full model

	Item	Valid N	missing	don't know	mean	sd	λ
	**Leadership**						
**1.**	Taking responsibility for the partnership	199	10 (4.6%)	9 (4.1%)	2.97	.78	.78
**2.**	Inspiring or motivating people involved in the partnership	199	11 (5.0%)	8 (3.7%)	2.81	.82	.82
**3.**	Empowering people involved in the partnership	200	10 (4.6%)	8 (3.7%	2.80	.76	.86
4.	Communicating the vision of the partnership	193	12 (5.5%)	13 (6.0%)	2.75	.78	.67
5.	Working to develop a common language within the partnership	191	11 (5.0%)	16 (7.3%)	2.61	.78	.72
6.	Fostering respect, trust, inclusiveness, and openness in the partnership	197	10 (4.6%)	11 (5.0%)	2.92	.90	.81
7.	Creating an environment where differences of opinion can be voiced	197	10 (4.6%)	11 (5.0%)	2.93	.83	.78
8.	Resolving conflict among partners	175	10 (4.6%)	33 (15.1%)	2.68	.80	.74
9.	Combining the perspectives, resources, and skills of partners	186	10 (4.6%)	22 (10.1%)	2.77	.73	.84
10.	Helping the partnership be creative and look at things differently	192	11 (5.0%)	15 (6.9%)	2.67	.84	.83
**11.**	Recruiting diverse people and organizations into the partnership	192	10 (4.6%)	16 (7.3%)	2.81	.82	.83
							
	**Efficiency**						
**12.**	How well your partnership uses the partners' financial resources.	179	39 (17.9%)	n.a.	2.64	.88	.71
**13.**	How well your partnership uses the partners' in-kind resources	191	27 (12.4%)	n.a.	2.82	.88	.84
**14.**	How well your partnership uses the partners' time.	195	23 (10.6%)	n.a.	2.50	.80	.72
							
	**Administration and Management**						
**15.**	Coordinating communication among partners	186	12 (5.5%)	20 (9.2%)	2.63	.69	.76
16.	Coordinating communication with people and organizations outside the partnership	158	16 (7.3%)	44 (20.2%)	2.36	.65	.56
**17.**	Organizing partnership activities, including meetings and projects	188	14 (6.4%)	16 (7.3%)	2.85	.73	.81
18.	Applying for and managing grants and funds	142	14 (6.4%)	62 (28.4%)	2.80	.88	.51
19.	Preparing materials that inform partners and help them make timely decisions	172	14 (6.4%)	32 (14.7%)	2.53	.79	.78
20.	Performing secretarial duties	162	14 (6.4%)	42 (19.3%)	2.73	.83	.69
21.	Providing orientation to new partners as they join the partnership	137	16 (7.3%)	65 (29.8%)	2.63	.77	.78
**22.**	Evaluating the progress and impact of the partnership	170	14 (6.4%)	34 (15.6%)	2.59	.79	.77
**23.**	Minimizing the barriers to participation in the partnership's meetings and activities	171	14 (6.4%)	33 (15.1%)	2.53	.83	.77
							
	**Non financial resources**						
**24.**	Skills and expertise	174	13 (6.0%)	0	2.44	.62	.86
**25.**	Data and information	167	16 (7.3%)	0	2.31	.68	.82
**26.**	Connections to target populations	165	17 (7.8%)	0	2.35	.77	.80
27.	Connections to political decision-makers, government agencies, other organizations/groups	142	13 (6.0%)	0	2.09	.78	.51
28.	Legitimacy and credibility	173	13 (6.0%)	0	2.59	.58	.80
**29.**	Influence and ability to bring people together for meetings and activities	185	13 (6.0%)	0	2.34	.64	.88

Notes; items in bold are included in the short version; λ = factor loadings on the intended factor.

### Confirmatory Factor analysis with 29 items

All items had factor loadings above 0.60 on the intended factor except for item 16 (0.56), item 18 (0.51) and item 29 (0.58) (see Table 2 last column). The indices of model fit also showed that the model fit was sufficient (see Table 3 model 1). The significant Normal Theory Weighted Least Square χ^2 ^statistic is not surprising given its sensitivity to sample size; it was 1627.616. The RMSEA was below cut-off value and acceptable. IFI value was 0.99 and well above cut-off value of .95 and SRMR was well below cut-off value of 0.08. All indices indicated that the model was acceptable, but left room for shortening.

**Table 3:** Model fit of the full and short models

On imputed data (n = 218)	Χ^2 ^(p)	RMSEA	IFI	SRMR
Model 1: 29 items on imputed data	1627.616 (0.0)	0.0524	0.988	0.0662
Model 2: final short version 15 items	432.634 (0.0)	0.0608	0.988	0.0638
**Listwise deletion 29 items (n = 68)**				
Model 1: 29 items	97.977 (0.0)	0.0603	0.949	0.0436
Model 2: final short version	170.213 (0.0)	0.0499	0.992	0.0761
**Listwise deletion 15 items (n = 111)**				
Model 2: final short version	300.730 (0.0)	0.0561	0.990	0.0719

Notes; model 1 = Confirmatory Factor Analysis on full version with 29 items; Model 2 = Confirmatory Factor Analysis on short version with 15 items

### Item reduction analysis

Following the factor loadings, modification indices and checking the internal consistency of each subscale, the stepwise procedure resulted in elimination of items in the following order: 27, 16, 18, 4, 20, 5, 8, 19, 21, 7, 6, 9, 10 en 28. The final short version consisted of 15 items with three or four items for each subscale. Item reduction could be done without loss of model fit. The overall fit of this final model was comparable with the model fit of the full version (Table 3 model 2). Due to a decrease in the number of estimated parameters, the Normal Theory Weighted Least Square χ^2 ^significantly decreased to 432.634, RMSEA was around cut-off and still acceptable. The value of IFI remained 0.99 indicating that the specified relations between variables are well supported by the data. The SRMR index decreased to 0.0638, which is still below the cut-off point of 0.08 and indicates that the global fit of the overall model is good. The final short model on imputed data resulted in comparable factor loadings.

Rerun of the full model and item reduction analysis on the non-imputed dataset with N = 68 resulted in good fit indices and exactly the same order of items that were eliminated.

### Internal consistency and inter-correlations

Internal consistency as represented by Cronbach's alpha ranged from acceptable for the 'efficiency' subscale to excellent for the 'leadership' subscale (Table 4). Also the correlations between the full original subscales and short subscales are good ranging from .92 to 1.00, indicating acceptable coverage of the original sub dimensions. The four subscales were significantly and positively correlated, indicating conceptually related subscales.

**Table 4:** Scale characteristics and inter-correlations of the shortened subscales (n = 218)

	Items short version	Cronbach's alpha	original full scale	scale mean (sd)	inter-item correlations range	1	2	3
1. Leadership	1, 2, 3, 11	.87	.92**	2.79 (.67)	.56-.73			
2. Efficiency	12, 13, 14	.75	1.00**	2.60 (.67)	.44-.53	.47**		
3. Administration and Management	15, 17, 22, 23	.83	.91**	2.56 (.61)	.50-.60	.63**	.51**	
4. Non financial resources	24, 25, 26, 29	.84	.96**	2.29 (.52)	.50-.62	.51**	.52**	.55**

** p < 0.01 (1-tailed)

### Convergent validity

Data on convergent validity of the original PSAT are presented in Table 5. To estimate convergent validity of the instrument we looked at correlations between partnership functioning and the areas that are known to be related to synergy: (1) effectiveness of the partnership's leadership; (2) efficiency of the partnership; (3) effectiveness of the partnership's administration and management; and (4) sufficiency of the partnership's resources. Results show that all dimensions of partnership synergy are positively related to synergy (all *p *≤ 0.001). We investigated disease-management partnerships and expected that higher levels of partnership synergy would be related to higher levels of chronic illness care delivery [23]. The ACIC measures chronic illness care [19], therefore, we additionally analyzed relations between partnership dimensions with the ACIC to verify convergent validity. The results show that all dimensions of partnership are positively related to chronic illness care (all *p *≤ 0.001). These values indicated convergent validity. The relative weights and significance levels of the PSAT-S are similar to the original PSAT (Table 5). Again, all partnership dimensions are positively related to partnership synergy and chronic illness care (all at *p *≤ 0.001). Results showed a strong relationship between disease-management partnership dimensions, synergy and effectiveness in chronic-illness care delivery. The advantages achieved by partnerships with high levels of synergy are likely to enhance partnership effectiveness in chronic care delivery.

**Table 5:** Correlation analyses of the partnership dimensions (original instrument and short version) with partnership synergy and assessment of chronic illness care (n = 218)

Partnership dimension	Leadership	Efficiency	Administration	Resources
*Original instrument*	*R*	*r*	*r*	*R*

Partnership synergy	0.622***	0.512***	0.535***	0.596***
Assessment of Chronic Illness Care (ACIC)	0.453***	0.339***	0.360***	0.466***
*Short version*				
Partnership synergy	0.609***	0.512***	0.513***	0.599***
Assessment of Chronic Illness Care (ACIC)	0.433***	0.339***	0.329***	0.447***

*** *p *≤ 0.001

## Discussion

This study aimed to validate the PSAT in various disease-management partnerships. Our results showed that the PSAT is a valid and reliable instrument to measure partnership synergy and its dimensions. The advantages achieved by partnerships with high levels of synergy are likely to enhance partnership effectiveness in prevention and health promotion that have been identified by other investigators [24]. The PSAT scores assess partnership's strengths and weaknesses in areas that are known to be related to synergy: (1) effectiveness of the partnership's leadership; (2) efficiency of the partnership; (3) effectiveness of the partnership's administration and management; and (4) sufficiency of the partnership's resources. This information can help partnerships identify in which areas they are doing well and in which areas the partnership needs to improve the collaborative process [7,8].

We investigated individual assessment of each professional participating in the disease-management partnerships. Analyses could also be performed at the partnership level taking into account the hierarchical structure of the data for individuals nested within partnerships. As there is the potential for considerable variation within partnerships and since the main purpose of our study was to compare the psychometric properties of the PSAT in disease-management partnerships, we performed confirmatory factor analyses on the individual level. Ignoring the hierarchical structure of the data may lead to a worse fit of the model. The factor loadings found with the two methods (individual versus team level) will however be similar in value [25,26].

The cumbersome length of the PSAT led us to perform an item reduction analysis and develop a short version (PSAT-S), which we later showed was reliable and valid. The results of the confirmatory factor analyses revealed good indices of fit with the PSAT-S. As indicated by the high reliability coefficient, the scale showed good internal consistency. In case the original PSAT is considered too lengthy, the PSAT-S is thus a good alternative. We found support for convergent validity of the original PSAT and PSAT-S through high correlations between the partnership dimensions, partnership synergy and chronic illness care.

Several psychometric properties could not be evaluated in this study and thus remain undefined, such as its predictive value. The instrument's sensitivity to change requires further investigation. As does its sensitivity to identify changes in the domains of partnership functioning and synergy for different partnerships. We also recommend testing the English version of the PSAT-S in other countries to ensure international validity. In addition to further research on the predictive value of the PSAT and PSAT-S, the presence of a control group (or control sites) would have also strengthened our conclusions. While it is possible that completing the PSAT or PSAT-S could act as an intervention based on the incidental education awarded by the survey itself, we do not think it likely given the difficulty in producing partnership synergy. Response rates varied widely (35% to 100%). We do not know how the results might have varied with a better response. Finally, we did not include patients as partners. Future research is necessary to investigate patients' perspectives of partnership functioning and use of the PSAT or PSAT-S among patients as well as professionals.

## Conclusions

With these shortcomings in mind, we conclude that the psychometric properties of the PSAT and the PSAT-S are good and the PSAT-S is a promising alternate instrument to evaluate partnership synergy and its dimensions.

## Abbreviations

PSAT: Partnership Self-Assessment Tool; PSAT-S: Partnership Self-Assessment Tool Short version; ZonMw: Netherlands Organisation for Health Research and Development; COPD: chronic obstructive pulmonary disease; CCM: Chronic Care Model; ACIC: Assessment of Chronic Illness Care; RMSEA: Root Means Square Error of Approximation; SRMR: Root Means square Residual; IFI: Incremental Fit Index

## Competing interests

The authors declare that they have no competing interests.

## Authors' contributions

AN drafted the design of the study. AN and JC were involved in data collection and all authors performed the statistical analyses. JC drafted the manuscript, MS and AN helped drafting the manuscript and contributed to refinement. All authors read and approved the final manuscript.
